# Improving Clinical Skills in Psychiatry Using Online Simulation

**DOI:** 10.1192/bjo.2022.149

**Published:** 2022-06-20

**Authors:** Sonya Rudra, Abigail Swerdlow

**Affiliations:** East London NHS Foundation Trust, London, United Kingdom

## Abstract

**Aims:**

The COVID-19 pandemic impacted medical education with teaching moving online. The aim of this study was to evaluate whether online simulation is an effective tool for the delivery of student psychiatric clinical skills teaching. This has important implications for the future planning of psychiatric clinical skills teaching.

**Methods:**

162 students were divided across nine online psychiatric simulation sessions held over a four month period. The sessions lasted 3.5 hours and consisted of three simulated scenarios with a professional actor and experienced facilitator. Students were asked to rate: confidence in taking a psychiatric history, conducting a mental state exam, formulating treatment plans, conducting risk assessment, assessing capacity and communicating with patients in psychiatry. Confidence ratings were completed pre and post session on Likert scale (1 = least confident, 10 = most confident). Students were also given the opportunity to provide qualitative feedback after the sessions. The study was conducted with permission from Associate Dean for Undergraduate Teaching and QMUL Centre Lead for Psychiatry.

**Results:**

137 (92.7%) of students attending the workshop completed pre-session questionnaire and 122 (82.4%) completed post-session questionnaire. 95.1% students rated workshops as good/very good. Pre and post confidence comparisons showed significant increases in average confidence for all questions from pre (M = 5.1, SE = 0.2) to post (M = 7.1, SE = 0.2), t = 10.7 p < 0.001. Paired t-tests were used to compare average pre and post-session results for individual questions from the same session. All questions showed significant increases in scores. Qualitative feedback indicated that students valued the opportunity to practice, obtain feedback and requested more sessions.

**Conclusion:**

Results show significant increases in confidence in psychiatric clinical skills using online psychiatric simulation. This supports our hypothesis that online simulation is an effective tool for delivery of student psychiatric clinical skills teaching. Students may benefit from online simulation increasing their confidence prior to attending psychiatric placements. This teaching method will also provide an additional method for practising clinical skills with increasing student numbers and demands on psychiatric placements. It therefore has important implications in the future of psychiatric education and could be adapted for use across clinical years and medical schools.

